# Orders- Versus Encounters-Based Image Capture: Implications Pre- and Post-Procedure Workflow, Technical and Build Capabilities, Resulting, Analytics and Revenue Capture: HIMSS-SIIM Collaborative White Paper

**DOI:** 10.1007/s10278-016-9888-7

**Published:** 2016-07-14

**Authors:** Dawn Cram, Christopher J. Roth, Alexander J. Towbin

**Affiliations:** 1 Department of Information Technology, University of Miami Health System, 1425 N.W. 10th Avenue, Miami, FL 33136 USA; 2 Duke Health Technology Solutions, Hock Plaza, 2424 Erwin Road, Durham, NC 27705 USA; 3 Department of Radiology, Duke University Hospital, 2301 Erwin Road, Box 3808, Durham, NC 27710 USA; 4 Department of Radiology, Cincinnati Children’s Hospital Medical Center, 3333 Burnet Ave, MLC 5031, Cincinnati, OH 45229 USA

**Keywords:** Imaging, Enterprise imaging, Clinical multimedia, Imaging workflow, Encounter, Encounters-based, Orders-based, DICOM, non-DICOM, Imaging informatics, Opthalmic imaging, Scope video, Clinical photography, Visible light imaging, Dermatology, Medical photography, XDS, XDS-i, Image capture, MWL, EHR, Enterprise archive, PACS, VNA, ECM

## Abstract

The decision to implement an orders-based versus an encounters-based imaging workflow poses various implications to image capture and storage. The impacts include workflows before and after an imaging procedure, electronic health record build, technical infrastructure, analytics, resulting, and revenue. Orders-based workflows tend to favor some imaging specialties while others require an encounters-based approach. The intent of this HIMSS-SIIM white paper is to offer lessons learned from early adopting institutions to physician champions and informatics leadership developing strategic planning and operational rollouts for specialties capturing clinical multimedia.

## Introduction

Images are being captured with increased frequency across specialties outside of radiology and cardiology throughout medical enterprises, including dermatology, ophthalmology, wound care, otolaryngology, and emergency departments [1]. Secure, centralized, and efficient image storage and management in these non-traditional environments is currently challenging [2–4]. Clumsy workflows proliferate across acquiring specialties [2, 5, 6]. In this paper, we identify benefits and challenges of defining the proposed categories of orders-based and encounters-based imaging, examine methods to manage, and suggest future prospects.

We define encounters-based imaging as being performed during a clinic visit or procedure when image content acquisition is not considered the purpose of the visit. There is usually no indication preceding the visit that imaging will be performed and imaging is at the sole discretion of the provider, as with dermatology photos. Often, encounters-based imaging compliments other clinical documentation, including progress notes, associated to the visit or procedure and may be referenced during follow up, surgery, or additional diagnostic exams.

Orders-based imaging acknowledges a more traditional approach [7]. An order placed in the source information system requests an imaging service to be performed, often by a different department or in a different physical location. An imaging department such as radiology receives and fulfills the requested order to answer the clinical question. In clinical reality, specialties such as obstetrics and cardiology employ this workflow commonly, though the imaging request may come from their own providers or from other specialties, and the imaging is often performed locally to the department. In these cases, the order placed is less intended to convey a diagnostic question and more to support downstream image storage and resulting workflows.

From a technical perspective, encounters-based imaging may be considered unsolicited, whereas orders-based imaging is considered solicited. However, this differentiation depends upon the methods employed by an organization to handle encounters-based images [7]. When an organization’s strategy requires the placement of an order from a source information system, such as an EHR, for the storage of images associated with the clinic visit, the imaging order is considered solicited. When an image management system delivers a record to the source information system of imaging performed without the source information system initiating the request, the imaging record is considered unsolicited.

## Goals

Regardless of the enterprise imaging strategy or individual workflows used, orders-based and encounters-based image storage and management workflows utilizing standards-based methods should provide the ability to:

- Identify all images associated with the care event, through the assignment of a unique study identifier,
- Associate images with a patient encounter, usually through a modality worklist or patient schedule,
- Manipulate image data, if required by the image use case,
- View images within the electronic health record (EHR) or directly from the EHR through a link associated with the note or report describing the visit where the images were obtained,
- Easily identify the type of imaging performed and the anatomical region through an EHR imaging description,
- Associate report or note describing the visit where the images were obtained with images in enterprise viewer, and
- Search necessary imaging metadata to serve business intelligence needs.

## Use Cases

Common clinical examples of orders-based image management workflows include procedures that are separately billable, having usually a technical and professional fee charged, such as MRI, CT, radiography, and echocardiography. There typically is a result separate from the originating clinical encounter; this result may or may not be necessary as part of reimbursement. Orders-based imaging workflows often require downstream system integrations for efficient resulting, such as to structured reports in echocardiography, electrocardiography, and obstetric ultrasound.

By contrast, several use cases exist today which are often first defined as encounters-based workflows for image management. Encounters-based workflows usually do not require a separate result and instead findings would be found in an associated encounter, procedure, or progress note, regardless of the visit being ambulatory, inpatient, or acute care. Medical photography is the most common use case for encounters-based workflows today. Often, but not always, dermatology, plastic surgery, emergency services, pathology, and endoscopy providers prefer encounters-based approaches. Recently, ophthalmology clinics are repositioning toward orders-based workflows due to reimbursement, report needs, and EHR integrations.

## Impact to User Workflow: Pre-Procedure

With respect to performing department user workflow, requiring an order for imaging performed ad hoc, acquired during clinic visits or associated with a procedure is not intuitive for clinicians and may be perceived as a responsibility not appropriate for a non-physician. For specialties like dermatology or wound care, multiple orders may be necessary to differentiate varied body parts and laterality. More specific localization information, like anatomical position, would require significant build within an EHR and may create system manageability issues. Difficulties in following moles, and other skin disorders/pathologies, without identifying more specific localization information may occur when several images of the same body part are acquired to document and follow multiple moles on the same patient. Placing orders for ad hoc imaging may require additional steps for the ordering clinician, such as opening an encounter only for imaging order placement. Multiple orders will also require clinicians to select a new modality worklist entry (DICOM MWL) for each order placed prior to acquisition and may inherently deter acceptance and compliance. For example, a patient on whom clinical photographs are taken in multiple anatomic locations, such as the right hand, upper back, and left ear, an orders-based workflow would require separate order placement for each set of images.

While specialties such as radiology may be able to offer orders combining multiple body parts which are often imaged together, this is not feasible for other specialties such as dermatology. For example, a CT of the chest, abdomen, and pelvis is routinely performed on patients based upon diagnosis protocols, whereas in dermatology, the body parts imaged are rarely indicated prior to the clinical visit and may be one of thousands of possible combinations. Although the ability to automatically spawn orders from an order set, or upon scheduling a clinic visit, is possible, implementing this build with encounters-based imaging may require a more generic description as the clinic visit will usually not contain indicators of the imaging to be performed. Since encounters-based imaging follows an ad hoc workflow, implications to post-procedure workflows may be noted, including cleanup of unused orders.

Encounters-based imaging workflows usually involve manually typing name and medical record number into the modality before images are taken. While this may take more time than entering the order and choosing the order at the modality, these steps are often done by non-providers in parallel with other patient care events by the provider. Limitations in resource availability to enter orders present administrative challenges. For example, staff may not be available or sufficiently trained to enter the order prior to acquisition and address subsequent order matching requirements and clean-up. Additionally, and perhaps of more significant concern, manual typing of patient demographics and exam information increases the probability of inaccurate identification and a higher incidence of data entry errors [8, 9].

For encounters-based imaging methods to be viable, mechanisms enabling the delivery of consistent image-specific information should be available on the capture device, including body part identification, acquiring specialty or an associated procedure description, and delivered as part of the image metadata. EHRs today are typically very familiar with orders-based approaches in radiology and cardiology and have components to accommodate those workflows; encounters-based workflows vary considerably and are generally not as well integrated with EHRs. The most notable considerations and implications of orders-based and encounters-based approaches before imaging are outlined in Table 1.

**Table 1:** Pre-procedure differentiator

Key differentiators: pre-procedure	Orders-based image management	Encounters-based image management
EHR build required	Large, including orders build and procedure dictionary maintenance concerns	Medium, including accommodation of new and varied workflows
Order placement during encounter scheduling	Clinically relevant order may drive downstream resources and workflows	Resource and workflow automation not possible
Order placement during encounter pre-procedure	Required, intrusive on workflow if done by provider or staff	Not required
Defining body part or procedure before imaging performed	Inherent in the order	Not readily available for most capture devices and applications
Defining laterality and specific anatomical position	Inherent in the order	Not readily available for most capture devices and applications
Clinical workflow for encounter-based imaging specialties	Challenging for clinicians and may impact acceptance and compliance with workflow	More intuitive, but if goals cannot be met will impact historical searches, analytics and subsequent viewing

## Impact to User Workflow: Post-Procedure

Major benefits associated with using an orders-based workflow include EHR users often find it permits better searchability and content relevance and is widely supported by image management systems and EHRs. A user may know if a given line item entry in the EHR pertains to their information need, or not, based on the order description and without opening the hyperlinked images. For example, a knee surgeon searching for scope camera images may be interested in knee scope images EHR entries, and will happily bypass elbow scope images. In contrast, encounters-based workflows may result in non-standard or generic imaging descriptors in the EHR.

Entirely orders-based workflows do have downsides after the procedure. Reconciliation workflows must be developed for cases where orders are not placed before imaging is performed, as occasionally happens during emergency department FAST exams. Likewise, when a procedure is performed and an imaging order is placed, but images are lost or never obtained, a cleanup process for the order is necessary. This cleanup may come at the expense of the user who placed the order or the institution health information management department.

In encounters-based approaches, some image management archives can instantiate orders from the modality sending the images, such that images from a given modality are all assigned an identical procedure order. This workflow may have limitations with modalities that are shared between multiple clinical specialties (e.g., perioperative ultrasound), where a single order (e.g., “ultrasound images”) may not be relevant or intuitive to EHR end users. Some organizations may instead employ ADT to orders transformation logic, generating the accession number within an interface engine and delivering a generic procedure description. This workflow may result in multiple instances of the same generated order or multiple body parts mixed within a single instance.

Image display protocols and multidisciplinary relativity should also be considered. The body parts and procedure descriptions traditionally associated with orders-based imaging often drive the ability to identify priors of like content for automated viewing protocols. Encounters-based workflows may only provide a lengthy encounter number and generic study description, unusable for such purposes. When implementing encounters-based workflows and desiring multidisciplinary relativity, it is necessary to determine the capabilities available for associating body part and a detailed description with the acquired images. Generic descriptions such as clinical photo will not provide enough information for comparison internal to the department or external to other specialties. The most notable considerations and implications of orders-based and encounters-based approaches after imaging performance are outlined in Table 2.

**Table 2:** Post-procedure differentiators

Key differentiators: post-procedure	Orders-based image management	Encounters-based image management
EHR content searchability	Standard metadata, determined by order name	May be included in clinical note or only generically described
Image archive instantiated orders	Not required	May only include generic description of imaging performed i.e. Derm Photo
ADT to ORM transformation	Not required	Generic description and may result in multiple instances of same order in archive
Prior image relevancy	Metadata provides viewer points to define prior image relevance	Cannot be ascertained with generic image description

## Build and Technical Challenges

EHR vendors today may have limited capability to index and tie metadata to medical images, perform image lifecycle management functions, or share images outside of the EHR system. Document management systems may be functionally limited in relating priors for comparison, may not offer multidisciplinary relativity capabilities, and may not support video and associated audio. Thus, EHRs and document management systems can not be the single location for storage of all medical images today; a separate vendor neutral archive or picture archiving and communication system (PACS) remains necessary. However, EHRs or document management systems may be the single storage location for a given specialty within a health system if the images are not DICOM, if metadata indexing is not critical to that specialty, comparison across acquisition dates is not desired, availability of a continuous image record across specialties is not essential, or if resulting workflows heavily favor having result text and images consumed together clinically on a given user interface.

In EHRs, using an orders-based workflow requires orders creation and distribution to the lists of commonly requested or performed procedures of involved clinical staff. Providers may not want the extra responsibility of placing an order for the image-based workflow to support image storage. Most providers would prefer to encourage their staff to adopt the order placement similar to how they may encourage their in-clinic staff to populate an ultrasound modality with demographic metadata in preparation for image capture. Often, the non-provider staff that the provider would prefer place the order do not have clearance to place EHR orders because this level of system security would also provide them with access to order many unrelated procedures. Designing an imaging workflow must additionally consider the access securities of those who would use it.

Automated methods of spawning an image order from appointments, operative case requests, and procedure encounters may not be technically available in all systems. Automated methods may present further challenges in specialties acquiring clinical photos such as dermatology and emergency services, as the visit does not specifically identify the body region where photos may be acquired. Confusion with compliance, IT, or operations staff may also be noted, believing that the order to store images actually represents an order to perform a procedure; for example, a scope camera image storage order may be misperceived as the order to perform an endoscopic procedure.

The support for DICOM modality worklist capabilities, both on modalities and on image review software applications, among vendors outside of cardiology and radiology is inconsistent. Workgroups and development is ongoing in specialties such as ophthalmology and pathology [10]. For both encounters- and orders-based image capture and storage, modalities may not have DICOM licenses, DICOM store capabilities, adequate modality storage capabilities, or networking to support the workflow. Additional challenges exist where DICOM MWL and IHE profiles, including scheduled work flow [11], support order placers which do not exist in an encounters-based imaging workflow.

In an encounters-based image archive model, solutions are expected to interpret and utilize a wide array of ADT events including A01s (Pt. Admit), A04s (Pt. Registration), A02s (Pt. Transfer), and A10s (Pt. Arrived) [12]. Logic supporting workflows tied to a visit, such as inpatient movement/transfers throughout a hospital, are also required for distribution of patient demographics tied to the unit(s) where each device is used [13]. When images are stored to the archive, patient validation criteria can only be gainfully applied based upon an open encounter for that patient, as in the instance of storing scope video associated with an otolaryngology procedure using an encounters-based methodology. For outpatient workflows, the encounter, or attending, provider associated with an ADT event is included within the HL7 message and is fundamental in generating provider worklists within an image viewer [14]. Traditional PACS and vendor neutral archives (VNA) may not store physician data beyond the referring, reading, and occasionally ordering, provider.

In contrast with EHRs, many PACS do not support non-DICOM image storage and distribution, leading organizations to look to VNAs for Cross-Enterprise Document Sharing (XDS) content support or requiring the purchase of vendor-specific systems to manage content and output DICOM formatted image files to the larger, centralized archive [15]. Although non-DICOM clinical images and multimedia may be managed more effectively through XDS workflows, VNAs are lacking in image workflow management tools like exceptions handling and content validation, serving solely as storage repositories for XDS content and images [16]. Resolving challenges associated with specialties acquiring non-DICOM images still requires archive product development supporting encounters-based imaging if organizations are looking to manage visible light imaging without requiring orders, regardless of the format acquired or stored [17].

Depending on the method of storage, images from encounters-based workflows may be more difficult to locate or share outside of the institution. Some EHR and ECM systems have imaging content storage that does not easily accommodate record sharing or transfer of images outward. A more extensive image exchange discussion is detailed in the HIMSS-SIIM Joint Workgroup white paper “Considerations for Exchanging and Sharing Medical Images” [18]. The most notable EHR and technical architecture considerations and implications of orders-based and encounters-based approaches are outlined in Table 3.

**Table 3:** Build and technical challenges differentiators

Key differentiators: build and technical challenges	Orders-based image management	Encounters-based image management
Image archive	Outside EHR in VNA/PACS	Could be VNA/PACS or EHR
DICOM images	Commonly supported	Development opportunity
Non-DICOM images	Development opportunity	Development opportunity

## Impact to Analytics

Image metadata applied from an order offers easily searchable terms for query and analysis. Although available image metadata may include patient- and order-specific information, often encounter information is not consumed or stored in a PACS or VNA and remains unavailable unless managing analytics through a third party data warehouse. Visit-specific information such as admitting diagnosis compared with resulting diagnosis may be desired for research, peer review processes, and quantifying value. Orders-based workflows may employ questions suggested or required during the order process that may capture additional relevant information for analytics.

A compromise that sites employing encounters-based workflows may sustain is having inherently “dirty data” in exchange for fewer clicks. Currently, there is limited ability within encounters-based workflows for data/exam validation the way there is with orders validation. For example, user typos manually entered at the modality to associate images with a patient and encounter can result in downstream errors of image association within the chart. In this instance, images could be lost or be filed in the wrong patient’s imaging record. Metadata added during encounters-based workflows, which are isolated to the storage system, will also limit the ability to search using EHR level queries. The most notable metadata considerations and implications of orders-based and encounters-based approaches are outlined in Table 4.

**Table 4:** Analytics differentiators

Key differentiators: analytics	Orders-based image management	Encounters-based image management
Metadata search	Orders content and context metadata	Object context image metadata only
Metadata location	Partially EHR, partially storage	Storage
Metadata capture	Configurable suggested or required information within order	Speed of workflow often contrary to additional data collection

## Impact to Resulting

Results may take several forms, and often differ based on a given provider or specialty’s use of orders or encounters-based imaging. Results may simply be an unstructured image description or a reference in the patient’s clinic note incorporated into other diagnostic test results. The image result may consist of a discrete description with a standard or frequently used template format. The result may be fully structured text, fully unstructured, or a mix of the two.

It is important for some specialties to include textual descriptions of the findings on the image, and ideally have the two reference each other. For example, the description and the image of a rash are often very valuable together, and more difficult to understand separately. With a small number of images, the images and result may be easily linked in same categorical or tabular EHR location, and often consumed in the same graphic user interface at any moment.

However, some order-based imaging specialties, such as cardiology and radiology, have many images per exam and require dedicated image manipulation tools for image review. All images from these specialties cannot be efficiently incorporated into an EHR note. Thus, resulting orders-based exams with images is best done through either a hyperlink to the full set of exam images that opens a dedicated viewer, or only the most critical images from the examination should be incorporated with the exam text.

Encounters-based workflows can be supported if resulting is managed through the EHR/Information System. It is challenging to manage when using third party resulting products. Providing imaging results back to an EHR/Information System without an order is not currently supported, unless the archive is capable of storing and relaying the associated visit number. Although this can associate the result to the encounter, it may also create two unlinked records within the encounter; one for the image hyperlink and another for the result. Delivering the EHR result to an image archive may present additional challenges associating the result to the images acquired during that visit.

When implementing a workflow requiring order placement in addition to the actual procedure order, as with scope video associated with a surgical procedure, the procedure notes and result may appear in a separate location from the imaging. Requiring the placement of multiple orders against a procedural encounter should be carefully considered when designing EHR and imaging workflows. Further noted, EHRs are often limited in their approach to intuitively display reports and notes with the associated images, frequently storing them as separate documents in the EHR, electronic content management system (ECM), or PACS/VNA. The most notable considerations and implications of orders-based and encounters-based approaches for resulting are outlined in Table 5.

**Table 5:** Resulting differentiators

Key differentiators: resulting	Orders-based image management	Encounters-based image management
Method of resulting	Orders-based or clinic note	Clinical note only
Image incorporation with clinical text	Typically hyperlink only	Hyperlink or direct inclusion of images alongside clinical text
Delivery of result to image archive	Commonly available/utilized	Development opportunity

## Billing/Reimbursement Considerations

Reimbursement usually drives how organizations design their imaging workflow rather than the imaging workflow determining how billing will occur. For example, if some insurers require a separate report for US exams, while others allow the result to be interpreted within a procedure note, an organization will most likely require that all US exam results be interpreted as separate reports. Usually, a third party product is used to dictate the US reports and an orders-based workflow would then be necessary. In some cases where a separate result is not required, a technical fee, if billable, can be added as charge and the pro fee wrapped into the clinic visit.

Generally, the method of image storage and management does not impact billing and reimbursement, assuming the images are stored somewhere that can be accessed in the event of a billing audit. However, long-term image storage is often required by regulatory or accreditation agencies or by insurers for reimbursement. In some cases, insurers require submission of imaging performed prior to a procedure for reimbursement of the procedure, as with clinical photos required for reimbursement of a blepharoplasty. This storage may occur either with encounters-based or orders-based approaches.

## Future Opportunities

For specialties practicing purely visible light still imaging, handheld photography, or video, such as dermatology or plastics, translating clinical workflows into the EHR leaves for clumsy and click-heavy image management and exceptions workflows. The majority of high-resolution cameras used in hospitals today cannot accommodate a DICOM modality worklist or an EHR ADT feed to associate images with metadata or with a patient encounter. The smoothest workflow today for clinical staff in dermatology, wound care, plastics, and other specialties utilizing photos for clinical documentation and follow up lies in image capture and management without orders. Orders-based workflow in these specialties is simply time prohibitive, user-unfriendly, and should not be expected.

To better support orders-based imaging workflow, EHR vendors should facilitate development of order securities that permit placement of order “containers” for images to live under in the EHR to document care, but do not permit placement of diagnostic test orders to dictate care. Application of searchable metadata to the orders should be simple and available at the point of care. Developing efficient processes for automated order or order set placement by non-providers during close encounter workflows could make metadata association with captured images easier. Finally, EHR vendors should better facilitate orders-based workflow image consumption alongside clinical text.

To better support encounters-based imaging workflow device vendors of visible light technologies will need to establish standards-based methods to provide a patient schedule, pass constant values describing image content, and make associating the image metadata more user friendly and time efficient than non-provider clinic staff placing an order. Image management systems will also need to develop practical support for encounters-based workflows, which is scalable across service lines and includes storage and utilization of ADT and case scheduling (SIU) events, provides workflow management tools, and supports EHR integration in conjunction with order-based images.

Whether an organization implements an orders-based, encounters-based, or combined workflow, developing methods to dynamically incorporate select archived images within progress notes, encounter notes, or distinct reports can eliminate the need to copy/paste or import specific images to provide a more complete result.

## Conclusion

The goals of orders-based and encounters-based workflows are very similar, and ultimately amount to best patient care:

- Identify all images associated with the care event, through the assignment of a unique study identifier,
- Associate images with a patient encounter, usually through a modality worklist or patient schedule,
- Manipulate image data, if required by the image use case,
- View images within the EHR or directly from the EHR through a link associated with the note or report describing the visit where the images were obtained,
- Easily identify the type of imaging performed and the anatomical region through an EHR imaging description,
- Associate report or note describing the visit where the images were obtained with images in enterprise viewer, and
- Search necessary imaging metadata to serve business intelligence needs.

It is anticipated that traditionally DICOM-based diagnostic image specialties such as cardiology, obstetrics, and radiology will stay orders-based for the foreseeable future. At least today, organizations prioritizing imaging analytics and searchability of images within the EHR may choose orders-based workflows in many clinical areas to capture necessary metadata to drive business intelligence and care after the initial image capture encounter. In environments with evolving and quickly advancing imaging use cases and technology, such as ophthalmology, operative suite, scope camera, and pathology, the choice of orders-based versus encounters-based workflow remains unclear. Future development and innovation is ongoing.

Orders-based workflows, while more refined and technologically supported, may create clinical workflow challenges for certain specialties where the imaging is inherently encounters-based and unpredictable. Counter-intuitive image acquisition and storage methods may incur provider frustration and limited adoption.

Currently, encounters-based imaging workflows are minimally developed by medical imaging device and system vendors, and are sub-optimally accommodated by EHRs. Many of the visible light modalities primarily associated with encounters-based imaging lack standards-based acquisition and storage methods resulting in limited associated metadata, generic descriptors, and prevalence for manual entry errors, EHR searchability issues, and analytic impact.

When determining which workflow to implement, organizations should understand the benefits and impacts within their environment and be clear with industry partners on needed functionality, while accommodating the workflow that provides the best patient care for that use case. We recommend that the goals proposed within this paper additionally be considered to ensure content availability and value.
